# The Experience of Korean Nurses During the Middle East Respiratory Syndrome Outbreak

**DOI:** 10.1177/0894318417741119

**Published:** 2018-01

**Authors:** Sook-Bin Im, Steven L. Baumann, Mina Ahn, Hyunok Kim, Bock-Hui Youn, MinKyoung Park, Ok-Ja Lee

**Affiliations:** 1 Professor & Dean, College of Nursing, Eulji University, Daejeon, South Korea; 2 Professor, Hunter College of the City, University of New York, Williston Park, NY, USA; 3 Headnurse, Department of Nursing, Eulji University Hospital, Daejeon, South Korea; 4 Nurse, Department of Nursing, Eulji University Hospital, Daejeon, South Korea; 5 Team manager, Department of Infection control, Eulji University Hospital, Daejeon, South Korea; 6 Assistant Professor, Department of Nursing, Koje College, Geoje-si, South Korea; 7 Director, Suprigajung, Daejeon, South Korea

**Keywords:** critical care nursing, isolation, Middle East Respiratory Syndrome (MERS)

## Abstract

The authors in this article explore the experiences of eight South Korean nurses during an outbreak of the Middle East Respiratory Syndrome (MERS), which took place in the fall of 2015. These nurses were mandated to remain in isolation in an intensive care unit (ICU) dedicated to the treatment of the patients with the MERS virus for 7 days. Parse’s humanbecoming theory was used to frame the discussion. Three themes found in the nurse’s stories are discussed: feeling hopeless and cut off, feeling shame and overworked, and feeling pride in fulfilling a duty. The nurses discuss how they overcame the difficulties of their situation, which ultimately reinforced their identities as nurses.

Contagious infectious diseases remain a global health challenge and a threat to nurses and other healthcare workers everywhere. The outbreak of the Middle East Respiratory Syndrome (MERS) in South Korea in the fall of 2015 is only one of several outbreaks that have occurred in the past 10 years. The outbreak of the MERS in South Korea was believed to have been carried there by a 68-year-old Korean man who flew back to Seoul from the Middle East, where he had visited four countries. The outbreak in South Korea involved 186 cases with 36 deaths (Wikipedia, 2017). Containment of the virus required the isolation of 2,361 patients and hospital staff members. This public health effort was necessary because of the lack of an effective vaccine or other ways to immunize healthcare workers and the caregivers of those infected with the virus, which was the main threat from this particular coronavirus (Butler, 2015).

While the threat of the MERS spreading outside of the hospital setting, aside from informal caregivers who had direct contact with affected persons, was fairly low, there was considerable public anxiety and concern, in part because the Ministry of Health and Welfare of South Korea decided not to provide information about how many cases there were and where they were being treated. Media attention to the outbreak was considerable, which as will be seen below affected the nurses who were isolated and their families. These nurses were also not getting sufficient information, so they too had considerable anxiety and fear as rumors spread through social networks and social media. During the outbreak, persons who had been hospitalized or had visited someone in the hospital where patients with MERS were being treated had added concerns that they would not be accepted back into the hospital to visit or if they themselves needed medical attention (Ha-eun, 2015). In the context of this MERS outbreak and widespread apprehension, ICU nurses who had been in contact with confirmed cases were mandated to remain isolated, and they were not allowed visitors during the time they were caring for persons with MERS.

## The Stories of Nurses

Early in the outbreak, the general understanding about MERS in South Korea and elsewhere was limited; it had first been reported in 2012 (Wikipedia, 2017). So the ICU nurses were understandably anxious and fearful, as were their patients and their family members, who were prevented from visiting and given little information. It was the nurse’s experience of being isolated with limited information about the MERS outbreak while caring for persons with MERS, which is explored in this article. Eight nurses who were isolated for 7 days in the ICU volunteered to speak with the authors. The site of their employment and isolation was a hospital in Daejeon Province, South Korea, which was one of 15 hospital that had a least one patient with MERS (Wikipedia, 2017). All of the nurses were females between the ages of 25 and 42 years at the time of the outbreak and who had from 2-19 years of ICU experience. The nurses were interviewed in November of 2015, after their period of being isolated and working with the patients with MERS. The nurses were asked to talk about their experience of isolation due to MERS. Their comments were audiotaped, and the tapes were transcribed and became the basis of their stories, which are reported below.

### Seo-yun’s Story

Seo-yun is a 35-year-old married nurse who had 9 years of experience working in an ICU prior to the MERS outbreak. She said the isolation week was very difficult, because “we were not prepared” and “we lived in fear of contracting the disease.” Seo-yun went on to say, “I felt trapped, having to wear stuffy protective clothing, in which you would perspire and could not do anything about it.” She also said, she recalled the gazes she received from others and remembers being photographed, which made her apprehensive about what the newspapers were saying and if people would have recognized her as the nurse they saw on TV. She also recalled traumatic moments, like the time one of the patients with MERS died. She said everything that had been in contact with the patient was put into the body bag with the deceased patient’s body and taken to be cremated. She recalled the pity she felt for the person, and it made her reflect on the own life, how she had lived, how this event was going to change her life. “I said to myself I have to live better, be more mature, and I happy to be free.”

### Jin-woo’s Story

Jin-woo is a 35-year-old mother of two children who worked as a nurse in an ICU for 16 years at the time of the MERS outbreak. She said, “I recall the perspiration with the protective clothing and how hard it was to breathe with the masks. I also lost my appetite and began to feel weak.” She also recalled getting what seemed to be hundreds of phone calls that she had to try to answer even though she had dozens of tasks to do; she thought to herself, “I am just one body!” “I was so stressed, faced with my limitations, and isolated, not allowed to meet with my family or my children.” “They were also not allowed to go to school, which made them very upset.” There was also prohibition on visitors, so the families of the patients called and took their anger out on us, they said terrible things to us.” The more experienced nurses tried to support the younger ones; we cared for each other and became close. We just wanted to finish the situation well and do a good job. Focusing on this helped me forget how hard it was.

### Min-seo’s Story

Min-seo is a 34-year-old single nurse who had worked for 12 years in the ICU before the MERS outbreak. She wondered, “What would I do if I got infected with the MERS virus?” She admitted she was afraid and felt panicky. She described it as “being in a greenhouse during the summer.” She described the ICU like a battlefield that had been bombed. Like Seo-yun, she recalled the gaze of other staff members; she said they made her feel like she was “vermin.” She also recalled how hard it was for the families of the patients who were not allowed to visit their ill and in some cases dying loved ones. After it was all over, the families came to appreciate that we also were isolated and worked hard to comfort their loved ones; this made me happy I was a nurse.

### Seo-hyeon’s Story

Seo-hyeon is a 39-year-old married nurse and mother of two children with 16 years of experience of working in an ICU at the time of the MERS outbreak. She said, “We could not communicate with each other and there was little cooperation from the medical team.” She described being isolated as being in a vast wilderness, yet walled up in a tight space.” “It was a terrible hell.” She went on to say she felt shame because she was not able to maintain the standard and nursing principles she had been taught to keep, for example to prevent secondary infections. But paradoxically she said she felt pride looking back at the attitudes she had which helped her get through it.

### Min-seo’s Story

Min-seo is a 25-year-old single nurse who had 3 years of ICU experience at the time of the MERS outbreak. She said she recalled how bad she wanted to take a break and go outside, but they were not allowed to leave the ICU. She said meals were provided, and they could get some sleep, but when they woke up all they could do was work. She, like the other nurses, felt the rest of the hospital staff were keeping them back. She remembers one telling her “return the food tray after you disinfect it.” She said statements like this made her feel like she was the patient with an infectious disease. But she appreciated that she was not isolated alone, so she could talk to her coworkers and said they became close with the senior nurses, like they were her sisters. Min-seo said, “We took care of each other, and became friends.” She said they also felt close with the patients, forced to share in their isolation.

### Ha-eun’s Story

Ha-eun is a 34-year-old single nurse with 2 years of experience working in the ICU when the MERS outbreak occurred. She said, “We had to trace all visitors who might have been exposed to the patients with MERS infections.” Like Ji-woo, she recalled all of the telephone inquiries that she had to deal with as well as the heavy patient load. She recalls having to take her goggles off from time to time because she was feeling like she was becoming dehydrated. She also recalled getting her picture taken by reporters and hearing about the release of the news article about them. She said she felt very disappointed with the way they were treated but appreciated that her colleagues were going through the same, so they encouraged each other. Ha-eun said if she had been alone it would have been worse. As did the other nurses who spoke with the authors, she said to herself, “I am a nurse, and this is my job,” so she did the best job she could to complete her job assignments, which she was not ashamed of, even if “I was dying.”

### Ji-a’s Story

Ji-a is a 42-year-old single nurse who had worked in the ICU, then switched to work in the emergency department, but was back working in the ICU for the past 2 years before the MERS outbreak. She said, “The perspiration was pouring out all the time, it was so hot, that I was often dizzy, it was because of all of the protective clothing we had to wear.” She admitted that she felt depressed, thinking that her life was ruined. She recalled how the person who would give them meals or exchange linen did so from as long a distance as he or she could. She, like Min-seo, used the word “vermin” to describe how they were made to feel by others. She and all of the other nurses described that the sharing of this experience and the encouragement they gave each other led to developing close relationships. In her words, “We offered hope and cared for each other”; because they helped each other endure the difficulties, they shared intimacy. She recalls that she felt as if she was released from prison when the isolation was over. But now she thinks it was like it was a dream and not altogether bad.

### Da-eun’s Story

Da-eun is a 27-year-old nurse with 2 years of experience working in an ICU at the time of the MERS outbreak. She, as others have suggested, said, “I felt I was in prison, like in a concentration camp, I felt alone and hopeless.” She also admitted that she was afraid of contracting the MERS virus. She says she never wanted to feel that way again. “I could not breath because of all the personal protective equipment, it was like I as dying.” She also felt others were critical and gazed at her, as if she were vermin. She recalled feeling after it was over and the news and others told them they did a good job that they were being hypocritical. Da-eun shared in the other nurses’ experience that they were learning from each other, encouraging each other, “You can do it.” She even recalled that at times there was a cheerful atmosphere. She recalled that after it was all over, family began telling them that they were proud of what they had done, because it was so difficult. She said families gave her support.

## Discussion

Three themes can be seen in the nurses’ comments and stories. These themes are described here as feeling hopeless and cut off, feeling shame and overworked, and feeling pride in fulfilling a duty.

### Feeling Hopeless and Cut Off

Feeling hopeless and cut off reflects the nurses’ experience of not only being physically isolated and restricted from contact with others but also existentially isolated and imprisoned by fear, apprehension, and apparent disregard. Unable to take a break from their situation, even sleep and eating provided limited escape or distraction from their situation. Limited information, knowledge, and access to their families added to their sense of isolation. While only a few of the nurses specifically mentioned feeling depressed or hopeless, they all reported uncomfortable emotional states and altered sense of time passing, as shifts blurred with their prolonged hours in the ICU, as uncertainty about their future gave rise to pessimism. Unable to be with their families was particularly hard for the mothers of young children. The isolation experience by these nurses extended to their children as they were not allowed to attend school, which speaks to the widespread nature of the anxiety about the poorly understood virus. The television news coverage of the situation at the hospital contributed to the nurses’ isolation, as they feared they would be readily recognized and ostracized from their communities.

The second principle from the humanbecoming perspective (Parse, 2014), which can be used to better understand the stories of the nurses isolated because of the MERS outbreak, is the paradox of connecting-separating, along with revealing-concealing and enabling-limiting. Most of the nurses reported that the manner in which hospital staff threated them not only added to their feeling cut off but also was an experience of feeling betrayed, which in the humanbecoming theory (Parse, 2014) is part of the understanding of dignity, which goes along with reverence, awe, and shame.

This theme echoes historical outbreaks of deadly contagious infectious diseases that caught the science, healthcare agencies, and governments off guard and could only be contained by strict isolation, and branding persons with the disease as literally untouchables or, in more contemporary language, stigmatized. Other contemporary outbreaks such as Ebola, Zika, and West Nile remind us of our vulnerability as human beings and create risks for nurses. These diseases have also added considerably to the costs of healthcare and the stress of the work.

### Feeling Shame and Overworked

The second theme that can be found in the nurses’ comments and stories is identified here as feeling shame and overworked. Having to answer a high volume of phone calls, many of them with angry and frightened family members, while carrying out numerous tasks that demand considerable attention to detail and caution, for prolonged shifts, all while wearing personal protective equipment (PPE) and surgical N95 respirators, represented extremely demanding nursework, by anyone’s standards. This included caring for persons dying from their MERS infection and related organ failure (Al-Dorzi et al., 2016). The PPE equipment was estimated to weigh as much as 5 kilograms and took about 5 minutes to don and remove. Perspiration, dehydration, and difficulty breathing were hard for the nurses to avoid as they worked, and several suffered from dizziness and presyncopal episodes. The nurses were exhausted by the workload and long shifts. The use of PPE and N95 masks can be seen in humanbecoming theory as enabling-limiting (Parse, 2014), in that they allow nurses to work protected from unseen pathogens, while it makes doing even simple tasks more difficult. Not unlike firefighters, soldiers, astronauts, and deep-sea divers, protective equipment suits and masks become close companions and occasional life savers.

These South Korean ICU nurses reported that they felt shame of being stigmatized even while they were working heroically with considerable risk to themselves and their families. Several said they felt “the gaze of others” in the hospital, which made them feel like they were “vermin,” unwelcome creatures that could spread deadly diseases. They did not like getting their pictures taken, and their images were included on the nightly TV news and in the social media. They feared what this public disclosure and media attention would do to them and their families; they came to share their patients’ disgrace. Shame as mentioned above is a part of the humanbecoming theory (Parse, 2014) as part of the understanding of dignity, along with betrayal, reverence, and awe.

Military metaphors are often used in medicine and healthcare. Min-seo, for example, used such a metaphor to describe the ICU dedicated for persons with MERS as a “battlefield that had been bombed.” Not only were these nurses on the frontlines and in the trenches, they felt like they had become the enemy. Uninformed about what was going on by the government and isolated “like vermin,” they feared the worse. In the absence of effective vaccines or other ways to immunize healthcare workers, persons in close contact with those ill with the contagious infections must be considered a potential vector of the disease that must be contained. The problem is that the virus needs to be contained, but the affected persons feel they have to be contained, an object to be feared, and whose feelings and rights could be denied. This is what the nurses found difficult to accept, and it was a challenge for them to make sense of it all. Furthermore, their natural defenses were not trusted, they needed barrier protections from the contaminated and unclean. It is interesting to consider that, even in one of the most technological advanced cities in the world, Seoul, Korea, with a very well-educated healthcare workforce, science and knowledge remain too slow and limited to keep up with ever-changing threat from microscopic viruses and other infections that have been resistive to medication.

### Feeling Pride of Fulfilling a Duty

The last and final theme that can be found in the stories of the nurses is one of transformation. The nurses’ comments tell a story of nurses working together as dedicated and courageous employees. The newer nurses were grateful to have the senior nurses provide them with encouragement. Differences and inherent rivalries between nurses, at least during the crisis, evaporated in the face of the demands, tasks, and trying situation that preoccupied their attention. The comradery and shared close quarters fostered a new level of closeness as colleagues, which the nurses said contributed to their success and reinforced their view of themselves as nurses who fulfilled their duty. It was for them deeply rewarding. The closeness that developed while jointly working on what needed to be done also provided the nurses with hope, which offset the anxiety and depression they were experiencing.

In humanbecoming (Parse, 2014) language, these stories can be articulated as connecting-separating with the enabling-limiting of revealing-concealing. It also gives rise to the concept of the principle of powering, which is described by Parse (2014) as “pushing-resisting; affirming-not affirming; and being-nonbeing” (p. 36). In the context of South Korea, nurses overcoming difficulties and courageously facing threats gave rise to pride in knowing that they were fulfilling their duty. One of the nurses said, “I am a nurse! I did my best.” Another said everything about it “was not all bad,” and “we even shared a cheerfulness.” The nurses recognized their work had value, and despite the hardship and danger, they were happy to be nurses. In their words, “we finished this situation well.”

## Conclusion

Outbreaks of new and ever-changing contagious life-threatening viruses in the age of rapid international travel and large crowded urban areas remain a global health issue, which all healthcare workers need to remain vigilant for and cooperate with their nursing and hospital administrators and public health officials to do their best to contain. Beside nurses and other clinicians, hospitals and healthcare organizations, nations, and international nongovernmental organizations (NGOs) need to work together to be successful. Open information and honestly told stories, like the ones told above, are very important and should be taken seriously. The story of the outbreak of MERS in South Korean should help nurses appreciate the value of observing universal precautions and help them to take seriously routine practice of donning PPE and test-fitting N95 masks. Running mock exposure episodes and agency-wide simulations make sense to help nurses and other healthcare workers do what they have to do without undue anxiety or fears that can lead to mistakes and accidental exposure. Careful attention to infection disease updates and public health information is critical for all nurses and other healthcare workers.

On the personal and bedside level, the above narratives provide a story of anxiety bordering on public panic, which in this case gave way to a story of dedicated service of nurses who made considerable scarifying to fulfill their duty, working together to face a common threat or challenge. The three themes described above provide some understanding of what it was like being isolated and having to work wearing PPE and N95 masks for a week without a real break. These themes were described as feeling hopeless and cut off, feeling shame and overworked, and pride in fulfilling a duty. Humanbecoming perspective provides a framework that helps nurses make sense of such events and remain professional in difficult work situations. In this case, nurses working together ultimately reaffirmed their view of themselves as nurses, as they took care of MERS-infected patients in an ICU. Such self-affirmation is necessary when one considers that the general public and even some colleagues stigmatize nurses for doing what they do, and in some places, this stigmatization and discrimination extend to their families.
